# Alpha-Linolenic Acid Mediates Diverse Drought Responses in Maize (*Zea mays* L.) at Seedling and Flowering Stages

**DOI:** 10.3390/molecules27030771

**Published:** 2022-01-25

**Authors:** Xuejing Zi, Shiyong Zhou, Bozhi Wu

**Affiliations:** College of Agronomy and Biotechnology, Yunnan Agricultural University, 52 Fengyuan Road, Kunming 650051, China; zixuejing1994@163.com (X.Z.); zhoushiyong_ynau@outlook.com (S.Z.)

**Keywords:** long-term drought stress, widely targeted metabolome, transcription factor, α-linolenic acid

## Abstract

Water shortage caused by long-term drought is one of the most serious abiotic stress factors in maize. Different drought conditions lead to differences in growth, development, and metabolism of maize. In previous studies, proteomics and genomics methods have been widely used to explain the response mechanism of maize to long-term drought, but there are only a few articles related to metabolomics. In this study, we used transcriptome and metabolomics analysis to characterize the differential effects of drought stress imposed at seedling or flowering stages on maize. Through the association analysis of genes and metabolites, we found that maize leaves had 61 and 54 enriched pathways under seedling drought and flowering drought, respectively, of which 13 and 11 were significant key pathways, mostly related to the biosynthesis of flavonoids and phenylpropanes, glutathione metabolism and purine metabolism. Interestingly, we found that the α-linolenic acid metabolic pathway differed significantly between the two treatments, and a total of 10 differentially expressed genes and five differentially abundant metabolites have been identified in this pathway. Some differential accumulation of metabolites (DAMs) was related to synthesis of jasmonic acid, which may be one of the key pathways underpinning maize response to different types of long-term drought. In general, metabolomics provides a new method for the study of water stress in maize and lays a theoretical foundation for drought-resistant cultivation of silage maize.

## 1. Introduction

Drought stress (DS) is a vital abiotic stress which can cause serious damage to plants. It inhibits seed germination and plant growth rate, and cause wilting, premature senescence, and death [1,2]. Drought can also lead to changes in the composition and content of organic compounds and other substances in plants, such as starch [3], flavonoids [4,5], osmolytes proline [6], soluble sugar [7] and betaine [8], endogenous hormones abscisic acid (ABA) [9] and jasmonic acid (JA) [10], and nutrients (lignin [11], lipids [12]). Germination and early plant development are most sensitive to water stress [13]. In the context of global warming and frequent occurrence of extreme weather events, water shortage is becoming a major constraint to global maize planting and increasing of maize yield per unit area [14]. Crop yield and grain quality reduction caused by DS are the key problems to be solved urgently in scientific research. As one of the important grain crops, maize (*Zea mays* L.) is somewhat drought resistant, which is of great significance in ensuring good yields toward meeting the human food demand [15]. To some extent, the crop capacity to avoid drought stress has a significant linear relationship with grain yield and quality [16]. Plant responses to short-term DS include stomatal movement, physiological and metabolic changes, and accelerated development process [17]. Under water deficiency, maize accumulates different metabolites at different developmental stages. These metabolites have varied roles, which is the evolutionary manifestation of dynamic adaptation to environmental changes of maize and other species [18].

It is worth noting that maize under DS exhibits a very complex process of plant cell signal transduction [19]. Under drought, maize shows a series of reactions, including regulating osmotic pressure through the synthesis of sugars and free amino acids, activating anti-oxidative enzymes such as superoxide dismutase (SOD) [20] and catalase (CAT) [21], and antioxidants glutathione s-transferase (GSH-S) [22] and flavonoids [23] to remove reactive oxygen species (ROS) or control stomatal opening and closing through ABA hormone [24].

Water stress stimulates the activation of the metabolic regulatory network, and the upstream genes get expressed strongly. High-throughput sequencing technology has accelerated the research on DS responsive genes, and a large number of studies have been published on transcriptome sequencing. The drought-response gene *BdCIPK31* overexpressed in tobacco enhanced its drought resistance through the synthesis of encoded protein kinase CIPK [25].

When maize leaves are subjected to drought threat, stress response related genes are overexpressed in quantity, including the chloroplast-related heat shock protein HSP93-V [26]. Under drought conditions, *ZmMPKL1* overexpression caused stomatal enlargement, cell water loss and leaf wilting in maize seedlings, whereas knockout seedlings showed opposite phenotypes [27]. However, the variable cultivation environment conditions make the signal transduction pathway of plant cells increasingly complex and dynamic [28,29]. Widely targeted metabolomics has developed rapidly in recent years, allowing us to elucidate downstream processes in plant stress through different types and levels of small molecule compounds. This process is used with plant phenotypic and physiological data to reconstruct complex signaling pathways [30,31]. In DAMs detected in alfalfa plants under drought stress, it was shown that inositol and proline could improve the drought tolerance [32]. The metabolism of raffinose family oligosaccharides in maize is important in determining yield performance under DS. In particular, the levels of inositol and raffinose family oligosaccharides are markers of maize drought resistance; under normal irrigation, they are highly correlated with grain yield in DS [33]. *Arabidopsis thaliana* has also been shown to maintain lipid content and lipid composition stability under severe water loss conditions. The digalactosyl diglyceride (DGDG) synthase, monogalactosyl diglyceride (MGDG) synthase and unsaturated fatty acids in leaves increased under DS [34].

In the present study, maize plants were grown in pots in a greenhouse. Soil moisture was controlled by irrigation based on frequent measurements using a soil moisture meter. DS was imposed in seedling stage (2–10 leaves) and flowering stage (12-R1 silk-producing stage) for up to 15 days. The transcriptome and metabolome were separately determined in leaf samples with each drought treatment being finished. A multiple omics approach underpinned discovery of new drought response mechanisms in different developmental stages. This study provides a theoretical basis for silage maize drought-resistant cultivation technology.

## 2. Results

### 2.1. Phenotypic and Physiological Responses to DS Imposed in Different Growth Stages

**Figure 1:**
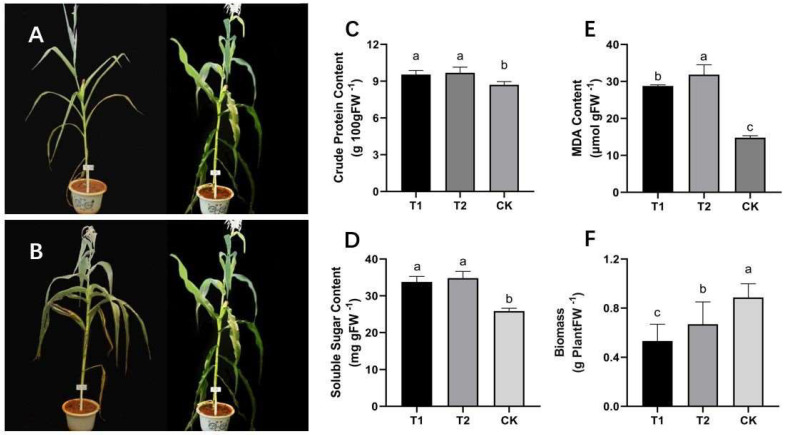
Phenotypic and physiological changes of control maize plants and those exposed to T1 and T2 water stress treatments. T1 and T2 represent long-term severe drought stress during the seedling and flowering stages, respectively, and CK is the conventional irrigated control. (**A**) phenotypes of drought treatment T1 and control CK in the seedling stage at harvest; (**B**) phenotypes of drought treatment T2 and control CK in the flowering stage at harvest; (**C**) changes in total protein content of the whole maize plants in each treatment; (**D**) changes in the soluble sugar content of treated maize leaves; (**E**) changes in the accumulation of malondialdehyde (MDA) in the maize leaves in each treatment; (**F**) comparison of the biomass of the whole maize plants at harvest. Different lowercase means values significantly different at *p* < 0.05. Each treatment has three replicates.

By limiting the irrigation of silage maize at seedling and flowering, the response model of silage maize to drought at different stages was explored. Regarding the plant morphology (Figure 1A,B), compared with the control, T1 and T2 treatments resulted in shorter plants with thicker stem, smaller leaf area and yellow curled leaves, which reflected the lack of nutrients and accumulation of flavonoids. Compared with CK, the plant biomass in T1 and T2 decreased significantly (Figure 1F). In T1 and T2 drought stress treatments, there was an increase in crude protein concentration (Figure 1C), likely due to the drought-altered gene expression. Plants in T1 and T2 accumulated more soluble sugars than the control plants (Figure 1D). In addition, drought induced membrane lipid peroxidation, and malondialdehyde (MDA) (Figure 1E), as the end product of this process, was also significantly increased in the T1 and T2 treatments.

### 2.2. Metabolomic Analysis during DS in T1 and T2

**Figure 2:**
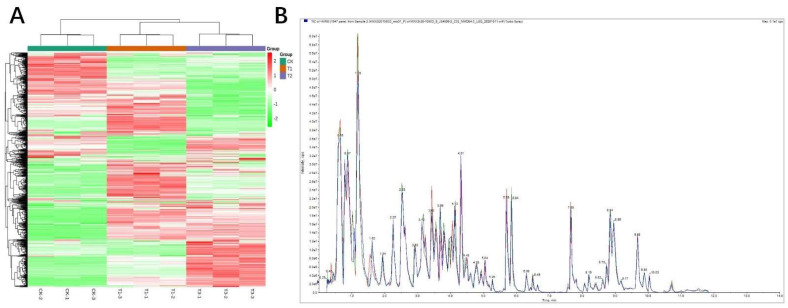
Cluster heat maps and total ions’ current chromatograms of control maize plants and those exposed to T1 and T2 water stress treatments. T1 (drought stress at seedling stage), T2 (drought stress at flowering stage), CK (conventional irrigation). (**A**) cluster heat map of all the metabolites in T1, T2 and CK; (**B**) overlay chromatogram of total ion current chromatograms (TIC) for mass spectrometric detection of quality control (QC) samples.

In this research, three treatments were involved, each represented by three biological replicates. The cluster heat map showed good repeatability of metabolites between groups in the identical treatment and significant differences in metabolites in different treatments (Figure 2A). By analyzing the overlay chromatogram of total ion current chromatograms (TIC) for mass spectrometric detection of various quality control samples, the results showed the chromatograms of TIC were highly overlapped (Figure 2B), which indicated that the signal stability was good when the same sample was detected at different times. Based on the UPLC-MS/MS detection platform and self-built database, 884 metabolites were detected, including flavonoids, phenolic acids, fatty acids, alkaloids, organic acids, amino acids, nucleotides and their derivatives, lignin and coumarin hormones, terpenes, and other metabolites (Supplementary Table S1, Figure 2A,B).

Comparing the T1 (Drought Stress at Seedling Stage) with the CK (Table 1), 302 differentially abundant metabolites were detected, of which 75 metabolites were downregulated and the remaining 227 metabolites were upregulated. Secondly, comparing the T2 (Drought Stress at Flowering Stage) with the CK, 294 metabolites differed in abundance, with 92 metabolites downregulated and the remaining 202 metabolites upregulated. There were 188 differentially abundant metabolites between T1 and T2, of which 113 metabolites were downregulated and 75 metabolites were upregulated.

**Figure 3:**
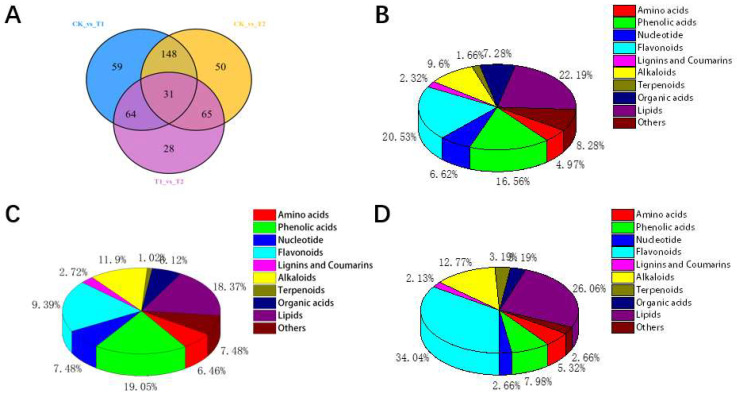
Venn diagrams and 3D pie charts of control maize plants and those exposed to T1 and T2 water stress treatments. T1 (Drought Stress at Seedling Stage), T2 (Drought Stress at Flowering Stage), CK (Conventional irrigation). (**A**) Venn diagram of differentially abundant metabolites between CK vs. T1, CK vs. T2, T1 vs. T2; (**B**) pie chart of the classification and proportion of the differentially abundant metabolites between T1 (drought treatment at seedling stage) and the control group, (**C**) between the T2 (drought treatment at flowering stage) and the control group, and (**D**) between T1 and T2 treatments.

Multiple comparison analysis (Figure 3A) showed there were significant differences in accumulation of metabolites between the drought treatments T1 and T2 and the control. The pairwise comparison showed the metabolites that differed among the groups were mostly flavonoids and fatty acids. Comparing T1 (drought at seedling stage) and control (Figure 3B), lipids (22.19%), flavonoids (20.53%), and phenolic acids (16.56%) accumulated significantly, whereas lignins, coumarins, terpenoids, amino acids, and other substances did not show obvious changes. Secondly, compared to the control (Figure 3C), in the drought-treated T2 at the flowering stage, flavonoids (19.39%), phenolic acids (19.05%), lipids (18.37%), and alkaloids (11.90%) accumulated significantly, whereas terpenoids, lignins, coumarins, and organic acids did not accumulate significantly. Finally, comparing the two drought treatments T1 and T2 (Supplementary Table S2), the main changes occurred in flavonoids (34.04%), lipids (26.06%) and alkaloids (12.77%), whereas the other substances did not change significantly (Figure 3D).

### 2.3. Pathways Enrichment Analysis of DAMs among CK, T1, and T2

**Figure 4:**
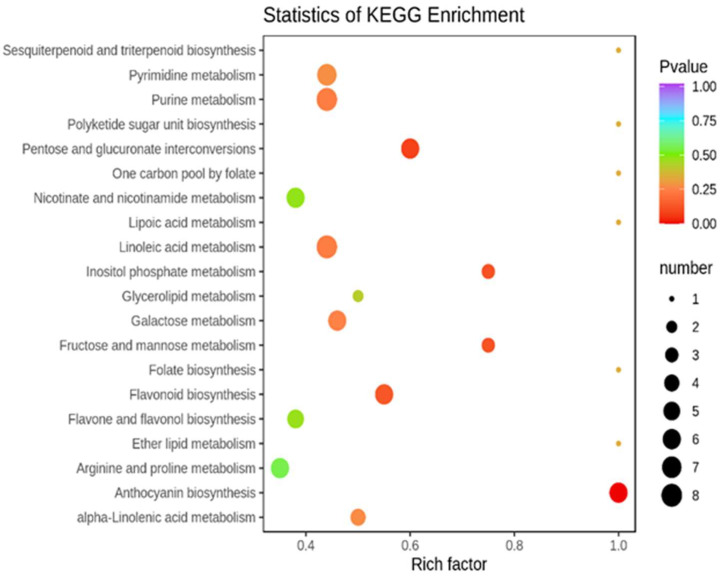
DAMs KEGG pathway enrichment between CK and T1.

**Figure 5:**
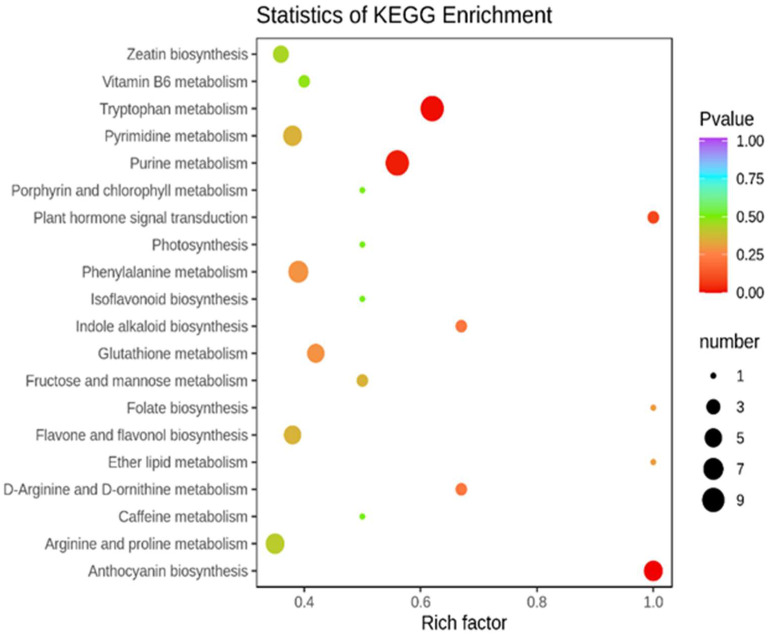
DAMs KEGG pathway enrichment between CK and T2.

**Figure 6:**
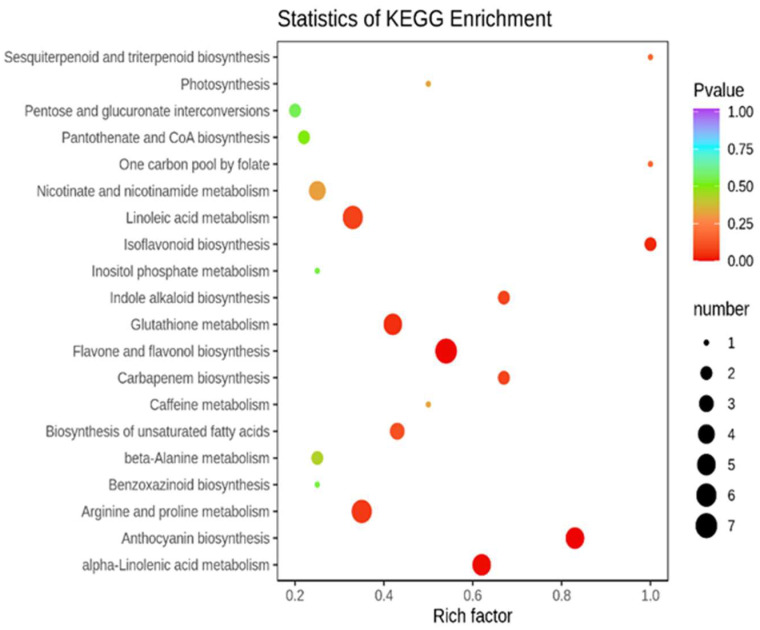
DAMs KEGG pathway enrichment between T1 and T2.

Compared with the control (*p*-value < 0.05), the treatment T1 (Figure 4) was mainly enriched in the anthocyanin biosynthesis, pentose and glucuronate interconversions and other pathways. The treatment T2 (Figure 5) was enriched mainly in anthocyanin biosynthesis, tryptophan metabolism, purine metabolism, and other pathways. To focus on the difference in DAMs under the two different drought forms of T1 and T2 (Figure 6), we found five pathways with a high degree of enrichment (*p*-value < 0.05); they were related to anthocyanin biosynthesis, flavone and flavonoid biosynthesis, alpha-linolenic acid metabolism, isoflavonoid biosynthesis, and glutathione metabolism.

### 2.4. Overview of RNA-seq Data for T1, T2, and CK

**Figure 7:**
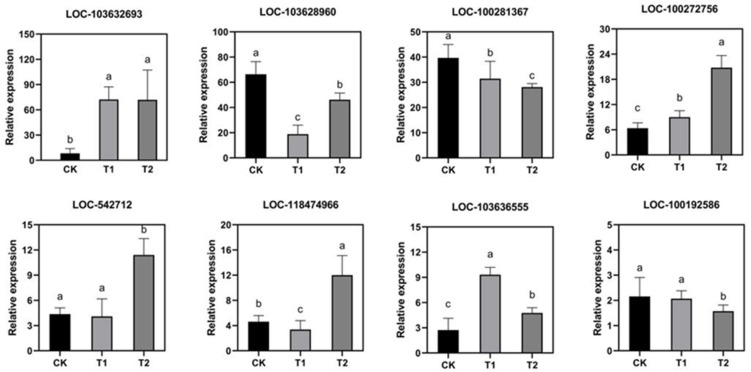
Verification of the reliability of the transcriptome data. Using Real-Time quantitative PCR, the expression levels of eight genes in T1 (drought at seedling stage), T2 (drought at flowering stage), and control CK were verified. The internal reference gene was *Actin-1*. *90A1: LOC-103632693*, *P47: LOC-103628960*, *OsCAD1: LOC-100281367*, *P450 73: LOC-100272756*, *F3H: LOC-542712*, *VvANR: LOC-118474966*, *CYP2: LOC-103636555*, *OsCAD1: LOC-100192586.* Different lowercase means values significantly different at *p* < 0.05.

In order to study the transcription levels of maize under different drought conditions, we used an Illumina HiSeq platform for high-throughput sequencing. Through RNA-seq, nine libraries were established (Supplementary Table S3). In general, 48,461,695, 47,733,287, and 46,614,559 clean reads were obtained for processing in T1, T2, and CK, respectively. The Q20 of all libraries was 97.86%, Q30 was 93.90%, and the GC content was about 55.01% (Supplementary Table S1). In order to verify the RNA-seq data, we selected eight genes for qRT-PCR verification (Figure 7), including *CYP2*, *P47*, *P450 73*, *OsCAD1*, *F3H*, *90A1*, and *VvANR*. These drought-related genes can be found in existing research. The real-time expression levels of the following eight genes were consistent with the results of transcriptome analysis.

### 2.5. Identification of Differentially Expressed Genes (DEGs) under DS

**Figure 8:**
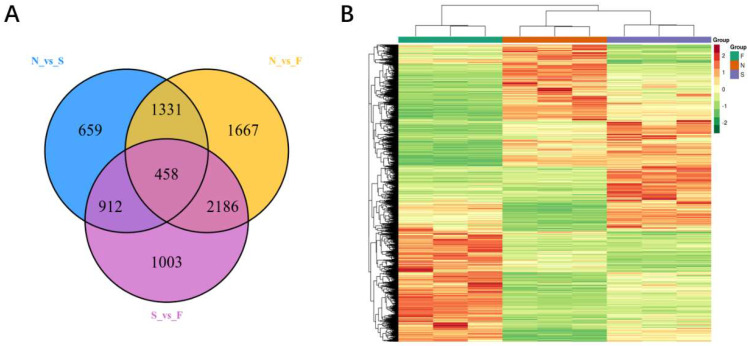
Venn diagram (**A**) and cluster heat map (**B**) of the differentially expressed genes. S (T1) represents long-term severe drought stress imposed at the seedling stage of maize, F (T2) represents long-term severe drought stress imposed at flowering stage of maize, and N (CK) represents control with conventional irrigation.

Compared with the control group, a total of 3360 differential genes were detected in the treatment with drought stress at the seedling stage of maize, with 1087 genes upregulated and 2273 genes downregulated. The treatment with drought stress at the flowering stage of maize resulted in 5642 differentially expressed genes, including 2621 upregulated and 3021 downregulated genes (Figure 8A). The cluster heat map showed good repeatability of metabolites between groups in the identical treatment and significant differences in metabolites in different treatments (Figure 8B).

### 2.6. The Annotation of Genes Differentially Expressed under DS Imposed in Seedling (S) and Flowering Stage (F)

**Figure 9:**
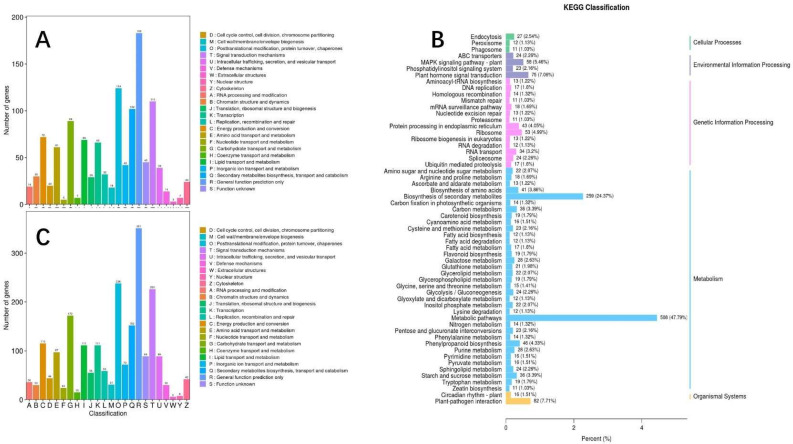
The KEGG classification of differentially expressed genes: between No drought & Seedling stage (**A**), between Seedling & Flowering stage (**B**), and between No drought & Flowering stage (**C**).

Through the KOG annotation, we can distinguish biological processes in which differentially expressed genes under the two types of drought treatments, S and F, were involved (Supplementary Table S4). As shown in Figure 9, under different drought treatments in maize, the DEGs were involved in “posttranslational modification”, “protein turnover”, “chaperones”, “signal transduction mechanisms”, “energy production and conversion”, “carbohydrate transport and metabolism”, “secondary metabolites biosynthesis”, “transport and catabolism”, and other processes (Figure 9A,B). However, there were treatment differences. Under the drought (F) treatment imposed during the flowering stage, 115 genes were involved in energy production and conversion and 111 genes in lipid transport and metabolism (Supplementary Table S5). Hence, maize may have different stress responses to drought depending on the growth stage in which drought commenced.

Comparing the T1 and T2 treatments, 4559 DEGs were detected (Figure 9C, Supplementary Table S6). The GO analysis of these DEGs showed that three main biological processes were enriched, including “biological processes”, “cell components”, and “molecular functions”. In terms of biological processes, these DEGs were enriched in secondary metabolite biosynthetic process. In terms of cellular components, DEGs were mainly enriched in DNA packaging complex. In terms of molecular function, they were mainly enriched in tetrapyrrole binding. The KEGG pathway enrichment analysis (Q value < 0.05) showed that a large number of DEGs were enriched in metabolic pathway, sphingolipid metabolism, carotenoid biosynthesis, phenylalanine metabolism, and galactose metabolism.

### 2.7. Analysis of DS-Responsive TFs (Transcription Factors)

Under the drought treatment during T1 stage, we identified 232 Transcription factors (TFs), involving 47 gene families (Supplementary Table S7). We selected 84 TFs with the gene expression level |log2FoldChange| > 2, including *AP2*/*ERF-ERF* (6), *bHLH* (8), *bZIP* (6), *MADS-MIKC* (7), *MYB* (9), *NAC* (9), and *WRKY* (5). Among them, six *NAC*, five *WRKY*, seven *bHLH*, and four *bZIP* were significantly upregulated, and six *AP2*/*ERF-ERF* were significantly downregulated. Under the drought treatment during the T2 stage, we identified 336 TFs, involving 44 gene families. The selected TFs (121 in total) included *AP2*/*ERF-ERF* (14), *bHLH* (9), *bZIP* (6), *MADS-MIKC* (7), *MYB* (14), *NAC* (10), and *WRKY* (10). Among them, eight *WRKY*, seven *NAC*, seven *MYB*, and seven *bHLH* were significantly upregulated, and eight *AP2*/*ERF-ERF* were significantly downregulated.

### 2.8. Correlation Analysis between DEGs and DAMs

In order to better understand the relationship between transcription factors and metabolites, the combined KEGG pathway analysis was performed on metabolite and gene data (Supplementary Table S8 and S9). T1 and T2 were enriched in 61 and 54 pathways, respectively, of which 13 and 11 were significant key pathways, most of which were involved in biosynthesis of flavonoids and phenylpropanoids, glutathione metabolism and purine metabolism. Interestingly, a comparison between T1 and T2 revealed significant differences in the α-linolenic acid metabolic pathway, with 10 differentially expressed genes and five differentially abundant metabolites in this pathway. Alpha-linolenic acid is an unsaturated fatty acid closely associated with plant antioxidant and jasmonic acid (JA) biosynthesis. We hypothesized that it may play different roles in response to drought stress imposed in different growth stages.

**Figure 10:**
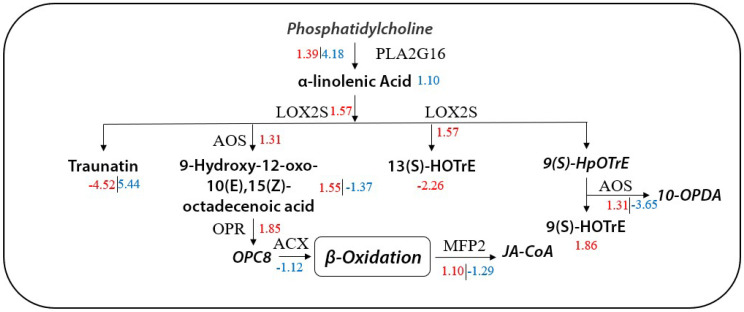
Metabolic network diagram of maize α-linolenic acid under T1 and T2 drought stress. Different nodes represent metabolites, and the lines represent the names of participating gene families. The red numbers indicate the upregulation or downregulation of metabolites or genes during T1. The blue numbers represent the upregulation or downregulation of metabolites or genes during T2. The fonts in italics represent unidentified metabolites, and some undetected compounds are not displayed.

**Figure 11:**
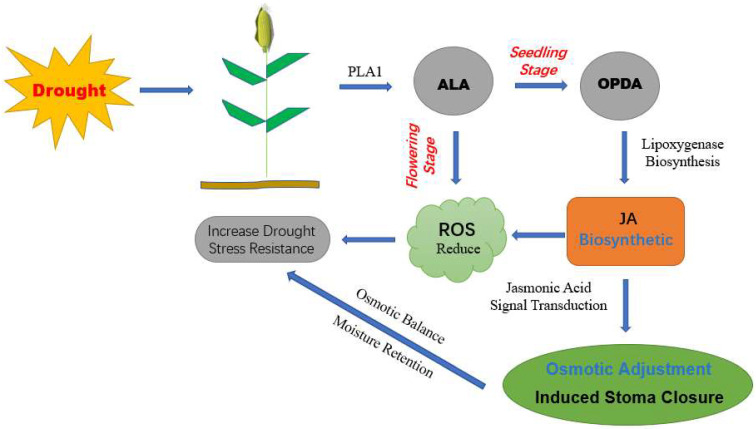
Schematic diagram of the mechanism of the response pathway of maize α-linolenic acid under drought stress at different developmental stages. PLA1: phospholipase a1-ibeta2; ALA: α-linolenic acid; OPDA: 12-Oxo phytodienoic acid; JAS: jasmonic acid; ROS: reactive oxygen species.

To test this hypothesis, we dissected the data further. According to the quantitative results presented in Table 2, compared with the control, the α -linolenic acid content of T1 and T2 increased by 21.9% and 40.6%, respectively. Furthermore, when T1 and T2 were compared regarding α-linolenic acid and its derivatives, four DAMs were upregulated significantly, and one DAM was significantly downregulated. Returning to the transcriptome data, we also found significant changes in DEGs between T1 and T2 (compared to the control) in the α -linolenic acid metabolism pathway, with 10 genes significantly upregulated in T1, and six genes significantly downregulated in T2. The key genes controlling JA synthesis (*ACOX3* and *LOC103653241*) were significantly upregulated in T1 and downregulated in T2. In addition, OPDA, a key product of JA synthesis, did not change significantly, but its relative content in T1 was higher than that in T2. Finally, we fully mapped DEGs and DAMs to this pathway and plotted the joint network of “α -linolenic acid metabolism” under T1 and T2 drought conditions (Figure 10). In addition, we drew a schematic diagram of maize α-linolenic acid response pathway under two drought treatments (Figure 11).

## 3. Discussion

By limiting the irrigation amount at the seedling and flowering stage, the response pattern of silage maize to drought was studied. Regarding the plant phenotype, T1 and T2 treatments showed smaller plant height and yellow and curly leaves, suggesting nutrient deficiency and accumulation of large amounts of flavonoids [34]. This is also reflected in the biomass difference of the whole plant. Compared with CK, the biomass of T1 and T2 decreased significantly, including the reduction of water content and assimilates [35]. DS disrupted the normal physiological process and metabolism of maize [36]. Our results showed that T1 and T2 resulted in an increase in crude protein, which was the result of differential gene expression under DS. Studies have confirmed the existence of 20 kD cyclophilic protein-like protein Sorg Cyp20 in sorghum leaves. After drought stress induction, the level of Sorg Cyp20 increased 3-fold [37]. On the other hand, plants in T1 and T2 accumulated more soluble sugars as regulatory substances to cope with osmotic pressure caused by insufficient water in the tissues [38]. Drought resulted in lipid peroxidation of cell membrane and accumulation of harmful substance MDA, which is a sign of cell membrane damage [39].

Based on the RNA-data, 1087 genes were upregulated under drought stress imposed at seedling stage (S) compared with the control group. When drought stress was imposed at flowering (F), 2621 genes were upregulated. A total of 47 gene families were identified under drought treatment in S period. The *WRKY* and *bHLH* families were significantly over-expressed at different DS stages, with the WRKY family considered to contain key genes of resistance to stress [40], which has been confirmed in Arabidopsis [41], rice [42], soybean [43], and millet [44]. The *WRKY57* and *WRKY20* showed especially significant changes.

Significant accumulation of lipids, flavonoids and amino acids was observed by comparing seedling drought with control using the metabolome data. Flavonoids, phenolic acids, lipids, and alkaloids were significantly accumulated under drought treatment at the flowering stage compared with the control. Flavonoids affect the color of plant leaves and stems [45], and their special phenolic hydroxyl and methoxyl functional groups give flavonoids and their congeners a strong oxidant activity to remove excess ROS [46]. Phenylpropane synthesis is the starting reaction of flavonoid synthesis, and flavonoid compounds are the branch pathway with the most metabolites in phenylpropane metabolic pathway [47,48]. The increased accumulation of anthocyanins, for example, can maintain higher antioxidant levels and provide a better balance between light capture and energy use [49].

Drought stimulates the operation of the cellular antioxidant system and produces a large number of secondary metabolites to improve the resistance [50], including peroxidase (POD), cytochrome p450 (CYP450), GSH-S, and other antioxidant enzymes, vitamins and alkaloids to maintain the REDOX homeostasis of cells [51]. First, at the flowering stage of maize (V12-R1, 12-leaf tassel stage to silking stage), under DS, photocontractual compounds produced by leaf cells and water are transferred to stamen and pistil, and a large number of free fatty acids and purines are transported to participate in grain development [52]. In order to promote the reproductive growth process and reduce the adverse effects of DS, and at the same time balance the reproductive growth, mature leaves and stems enter into a nutrient remobilization process, leading to premature senescence, such as linoleic acid, α-linolenic acid, and ricinoleic acid among free fatty acids [53]. Among DAMs, we found at least nine free fatty acid upregulated in the T2. Secondly, DS in the maize seedling stage (V2-V10, covering the third leaf from germination to the full extension of the tenth leaf) mainly affects the advancement of the vegetative growth process of maize, such as the reduction of photosynthetic performance and the slowdown of growth speed. It also brings changes in lipid metabolites [54,55].

By digging further, a separate comparison of T1 and T2 showed 188 DAMs, among which 113 DAMs were downregulated and 75 DAMs were upregulated. Multiple analysis showed that the changes of substance accumulation were mainly in flavonoids, lipids and alkaloids. Lipid changes caught our attention. Lipid metabolism plays an important role in plant response to DS [12]. Lipids decompose into free fatty acids under abiotic stress [47]. A large number of unsaturated fatty acids not only participate in the formation of grains but also contribute to strong oxidative stress resistance in crops.

KEGG pathway co-annotations showed that T1 and T2 caused enrichment in 61 and 54 pathways, respectively, among which 13 and 11 were significant key pathways involved mostly in biosynthesis of flavonoids and phenylpropanoids, glutathione metabolism, and purine metabolism. Similar results are also found in rice, soybean, and Arabidopsis [56]. Glutathione (GSH), an important member of the ROS-scavenging system, has been found to be involved in resistance to high and low temperatures, osmotic stress, and various other abiotic stresses [57]. Interestingly, between T1 and T2, and a total of 10 DEGs and DAMs were identified in a α-linolenic acid metabolic pathway. The same as recent research, changes in fatty acid patterns were observed due to reduced water supply, and polyunsaturated fatty acids such as linoleic acid and α-linolenic acid were produced in oilseed rape seeds [58].

α-linolenic acid in lipid metabolism can act not only as a strong antioxidant, but also as a precursor to the synthesis of JA, which acts as a signaling molecule to stimulate the downstream anti-stress response [59,60]. Numerous studies have indicated that the occurrence of JA is closely related to plant stress, including both biotic and abiotic stresses [61]. Salicylic acid is considered to be a signal molecule for plant resistance to pathogens, whereas JA and ethylene are signal molecules for plant resistance to insect feeding, mechanical damage, and abiotic stress [62,63,64]. In this study, the JA signaling pathway may be related to drought stress. Some published literature also proves this point. For example, a JA-mediated signal transduction pathway is associated with photosynthesis in rice under drought stress. The TFs involved are *JAZ*, *MYC2*, and *OsbHLH148* [65]. Induction of *FAD3* gene expression in *Arabidopsis thaliana* crown tumors provides a key to drought stress signaling, guaranteeing a long-term increase in the production of 18:3 FA (linoleic acid, α-linolenic acid, and their derivatives) [66]. A brief increase in JA concentration was detected in the roots of citrus under drought stress [67]. Under drought stress, exogenous JA increases the antioxidant enzyme activity of pearl chestnut seedlings, leading to a significant improvement in the plant defense system [68]. Similarly, prolonged water scarcity impedes the growth of *Arabidopsis thaliana*, thereby stimulating jasmonic acid signal transduction [69]. These examples clearly show that JA is closely related to DS on plants.

We give a hypothesis. α-linolenic acid mostly participated in the synthesis of JA during seedling drought and alleviated DS through JA signaling, while under DS at flowering, maize tended to use free fatty acids as antioxidant substances to reduce the damage. This may help us to further understand the drought response mechanism of maize.

## 4. Materials and Methods

### 4.1. Plant Materials and Drought Treatment

Two drought treatments T1 and T2 were set up in this study. T1 was treated with drought at seedling stage (V2–V10, covering the third leaf from germination to the full extension of the tenth leaf) for 15 days and T2 with drought at flowering stage (V12-R1, 12-leaf tassel stage to silking stage) for 15 days and a control CK (Conventional irrigation in the whole growth period). Two water gradients were set up referring to the national agricultural drought standard (GB/T 32136-2015, China). Severe drought had a field capacity (FC) range of 35–45%. Conventional irrigation had an FC range of 60–70%. The maize variety “Quchen 9” was provided by Agronomy College of Yunnan Agricultural University. The maize seeds were sown in mixed soil. Each pot was filled with equal amounts of 3 kg peat soil and 15 kg laterite. Its fundamental characteristics: pH is 7.12, organic matter content is 47.78 g/kg, and total nitrogen content is 2.10 g/kg. The plastic pots’ outer diameter is 41 cm; bottom diameter is 30 cm and its height is 25 cm. Each treatment had 20 pots as biological replicates. Pots were free draining and were watered with the same amount of water. Potted plants are placed in a greenhouse at Yunnan Dian-Tai Characteristic Agricultural Industrialization Engineering Research Center, Kunming, China. The indoor temperature was maintained at 22–32 °C.

**Figure 12:**
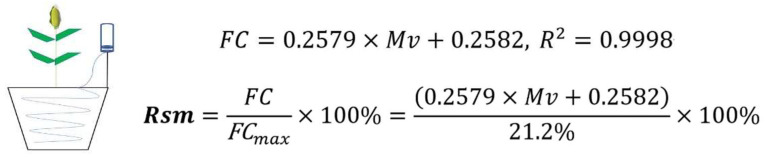
Water control device and first-order equations for soil volumetric and soil weight water content. FC means the field capacity; Mv is the volumetric water content; Rsm means the relative soil moisture; FCmax means the maximum field capacity. R^2^ is close to one, indicating a high degree of linear correlation.

Referring to the methods of some excellent research papers [70,71], we have improved the water control device. This device simply reduces the rate of flow of supplementary water while allowing for a more uniform distribution of water in the soil layer at all depths. For irrigation, we used a 750 mL plastic bottle, cut off the bottom, and drilled a 5 mm hole in the bottle top, inserted a transparent soft hose with a length of about 130.0 mm (outer diameter 5.0 mm, inner diameter 3.0 mm) and fixed it with adhesive. Wires were used to secure hanging plastic bottles (Figure 12). The hose was placed in another hose tracing the inner edge of the flower pot and inserted into the soil. The volumetric water content was measured every 24 h with a moisture detector (SPectrum, TDR-100, USA) according to the different treatment requirements. Soil volumetric water content (Mv) and field capacity (FC) values of the soil were measured in 20 sets of pots. Therefore, a first-order equation based on a direct linear relationship between Mv and FC was established as shown in Figure 12. The first order equation is found using the ordinary least square (OLS) method. We converted to FC by the first-order equation:

The range derived by the equation is as follows: severe drought with an FC range of 35–45% (Mv between 9.37–11.79%), conventional irrigation with an FC range of 60–70% (Mv in the range of 18.34–20.90%), and maximum field capacity of about 21.2%. According to different treatment requirements, every 24 h, there is real-time comparison of moisture detector reading, conversion of soil relative humidity monitoring and recording; and deficit irrigation is controlled using drip irrigation devices. TDR-100 was used to strictly control water supply and record at 10:00 a.m. every day. For CK, when its FC was below 60%, we promptly performed water supplementation, about 1000 mL every 2 days. For T1 and T2, we supplemented about 400 mL of water every 3 days during its 15 days of drought, and the rest of the stages were the same as CK. The self-made drip irrigation device was used for water replenishment. The hose flowed slowly to replenish water. The amount of water replenishment was calibrated with a measuring cylinder (1000 mL) and recorded. When the ordinary drought of basin soil drops to the standard range of soil water content, the water control in each growth period is continued. The amount of water replenished in the same treatment is different and dynamic. The total irrigation volume for the whole maize reproductive period was recorded for the various treatments as follows: CK with about 7.2 × 10^4^ mL; T1 with about 5.5 × 10^4^ mL; and T2 with about 5.6 × 10^4^ mL.

### 4.2. mRNA-seq Library Construction and RNA Sequencing

At the end of two drought periods of maize, we collected three leaves from the top of each plant, snap-froze them in liquid nitrogen, and stored the samples at −80 °C. There were three biological replicates for each treatment. MRNA sequencing extraction was performed. Ribosomal RNA was removed from total RNA to obtain mRNA. Subsequently, the RNA was fragmented into short fragments in a fragmentation buffer. First strand cDNA was synthesized using short fragment RNA as a template, and then two-strand cDNA was synthesized. Subsequently, double-strand cDNA was purified using AMPure XP Beads. Finally, PCR amlification was performed to obtain the final cDNA library [71,72]. After qualified database inspection, different libraries were pooled according to the target data amount, and sequencing was conducted using an Illumina HiSeq platform.

### 4.3. Sequence Reading and Analysis

Clean data were obtained by filtering, and sequence alignment was performed with the specified reference genome to obtain the mapped Data. The structural level analysis such as variable splicing analysis, new gene discovery, and gene structure optimization was performed [73,74,75]. Differential expression analysis, functional annotation, and functional enrichment were performed as described in [76]. Functional annotation of genes was performed using the KOG database (Clusters of Orthologous groups for Eukaryotic complete Genomes) of NCBI. R language is utilized for heat map making and data statistical analysis.

### 4.4. Sample Preparation and Metabolite Detection

Fresh maize leaves were frozen with liquid nitrogen immediately after sampling, and then stored in a −80 °C refrigerator (MDF-DU900VL-PC, London, UK). Biological samples were lyophilized under vacuum using a freeze-dryer (Scientz-100F, Ningbo, China), and crushed using a mill (MM-400, Retsch, Haan, Germany) with a zirconia bead for 1.5 min at 30 Hz. Solvent (1.2 mL of 70% *v*/*v* methanol) was added to 100 mg of lyophilized powder, vortexed six times (30 s each time with 30 min in between). The sample were left at 4 °C overnight. Following centrifugation (Centrifuge 5910 R, Eppendorf, Hamburg, Germany) at 12,000 rpm for 10 min, the extracts were filtered (SCAA-104, 0.22 μm pore size; ANPEL, Shanghai, China) before analysis.

The sample extracts were analyzed using an UPLC-ESI-MS/MS system (UPLC, SHIMADZU Nexera X2; MS, Applied Biosystems 4500, San Francisco, CA, USA). The analytical conditions were as follows: the column was Agilent SB-C18 (1.8 µm, 2.1 mm × 100 mm). The mobile phase consisted of solvent A (0.1% *v*/*v* formic acid in acetonitrile) and solvent B (0.1% formic acid in water). Sample measurements were performed with a gradient program that employed the starting conditions of 95% A, 5% B. Within 9 min, a linear gradient to 5% A, 95% B was programmed, and a composition of 5% A, 95% B was kept for 1 min. Subsequently, a composition of 95% A, 5.0% B was adjusted within 1.10 min and kept for 2.9 min. The flow rate was set as 0.35 mL per minute, and the column oven at 40 °C. The injection volume was 4 μL.

The LIT and triple quadrupole (QQQ) scans were acquired on a triple quadrupole-linear ion trap mass spectrometer, AB4500 Q TRAP UPLC/MS/MS System, equipped with an ESI Turbo Ion-Spray interface, operating in positive and negative ion mode and controlled by Analyst 1.6.3 Software (AB Sciex, Framingham, MA, USA). The ESI Source operation parameters were as follows: ion source, turbo spray; the source temperature 550 °C; ion spray voltage (IS) 5500 V (positive ion mode)/−4500 V (negative ion mode); ion source gas I, gas II and curtain gas were set at 50, 60 and 25 psi, respectively; and the collision-activated dissociation was high. Instrument tuning and mass calibration were performed with 10 and 100 μmol/L polypropylene glycol solutions in QQQ and LIT modes, respectively. The QQQ scans were acquired as multiple reaction monitoring (MRM) experiments with collision gas (nitrogen) set to medium. Declustering potential and collision energy were optimized for individual MRM transitions. A specific set of MRM transitions was monitored for each period according to the metabolites eluted within this period.

Metabolites were identified and quantified according to the following methods. Metabolite identification was based on the primary and secondary spectral data. The database used was metware database (MWDB). By comparison of the precursor ions (Q1), product ions (Q3), the retention time, and the fragmentation patterns with those obtained by injecting standards operating the identical conditions. Metabolite quantification was carried by MRM mode. The characteristic ions of each metabolite were screened through the QQQ mass spectrometer to obtain the signal strengths. Metabolites with significant differences were selected according to the variable importance in projection (VIP > 1) and fold change (FC ≥ 2 or ≤5).

### 4.5. Metabolite Analysis

The HCA (hierarchical cluster analysis) results of samples and metabolites were presented as heatmaps with dendrograms. The Pearson correlation coefficients (PCC) between samples were calculated by the cor function in R and presented as heatmaps only. Both HCA and PCC were plotted by R package heatmap. For HCA, normalized signal intensities of metabolites (unit variance scaling) were visualized as a color spectrum [77]. Significantly regulated metabolites between treatments were determined by variable importance in projection (VIP) ≥ 1 and absolute Log2FC (fold change) ≥ 1. VIP values were extracted from orthogonal projections to latent structures-data analysis (OPLS-DA) results, which also contained score plots and permutation plots, using R package MetaboAnalystR. The data were log transformed (log2) and mean centered before OPLS-DA. In order to avoid overfitting, a permutation test (200 permutations) was performed.

Identified metabolites were annotated using the KEGG Compound database (http://www.kegg.jp/kegg/compound/, accessed on 15 November 2021), and the annotated metabolites were then mapped to the KEGG Pathway database (http://www.kegg.jp/kegg/pathway.html, accessed on 15 November 2021). [78] Pathways with significantly regulated metabolites were then fed into MSEA (metabolite sets enrichment analysis); their significance was determined by hypergeometric tests’ *p*-values.

### 4.6. Determination of Soluble Sugars, Crude Protein, and Malondialdehyde Contents

The following physiological parameters were determined using dried leaf samples. Place the maize leaves in a brown disposable bag and deliver them in the oven (DHG-9053A) for dehydration. First, kill-enzyme torrefaction was carried out at 105 °C for 30 min. Next, change the temperature to 80 °C and continue for 24 h. Finally, dehydrated maize leaves are ground into powder operating a mill (LS-2500C, Linchang, China). The contents of soluble sugars, crude protein, and MDA in T1 and T2 leaves were determined. The content of soluble sugars was determined by the anthrone method [79]. Thiobarbituric acid (TBA) chromogenic reaction was used to measure MDA content to evaluate the peroxidation of membrane lipids [80]. Crude protein content was determined by an automatic Kjeldahl nitrogen analyzer [81]. The statistical analysis of physiological parameters was performed using GraphPad prime 8.0.1 software and using single-factor Duncan analysis.

### 4.7. Combined Analysis of Genes and Metabolites

The differentially expressed genes and differentially abundant metabolites in the same treatment were simultaneously mapped to the KEGG pathway map [82]. A histogram was drawn to show the degree of enrichment of pathways associated with both differential metabolites and differential genes.

### 4.8. cDNA Synthesis and qRT-PCR Detection

cDNA was synthesized using a one-step transcription kit using 2 µg RNA according to the manufacturer’s instructions (TransGen, Beijing, China). The RNA-seq results were used to verify 15 selected differentially expressed genes, including genes related to DS response. In the results, 8 genes with significant expression were selected and displayed. Primers were designed based on the Premier 5 and reference NCBI maize genome design. In QRT-PCR, a Bio-RAD (CFX 96) was used for real-time PCR detection, and the relative expression of target genes was calculated by the 2^−∆∆Ct^ method. The reference gene was maize gene Actin-1 [82].

### 4.9. Determination of α-Linolenic Acid 

Precisely weigh 5.00 g of samples, which are lyophilized and ground with liquid nitrogen, add 5mL of petroleum ether and mix. The mixture was extracted by an ultrasonic bath (Elmasonic S10H, Singen, Germany) at 50 °C for 30 min and repeated 3 times. The supernatant in the mixture was extracted using a 5 mL pipette and combined, and blow dried at 20 °C with nitrogen. Then, add 5 mL N-Hexane, 3 mL CH_3_OH-KOH (0.5 mol/L) to the oil extracted in the previous step, shake and mix, methylated in the oven (DHG-9053A, Shanghai, China) at 60 °C for 30 min, remove and centrifuge (Centrifuge 5910 R, Eppendorf, Germany) at 4000 r/min for 10 min. Finally, take all the supernatant and blow dry at 20 °C with nitrogen, then dissolve and fix the volume with 0.5 mL N-Hexane, shake, and mix with a 0.22 μm filter membrane, to be measured.

An Agilent series capillary gas chromatograph equipped with a flame ionization detector at 250 °C (GC7890, Agilent Tec, Wilmington, DE, USA) was used to profile the fatty-acid methyl esters. Alpha-linolenic acid was separated on a DB-FFAP capillary column (30 m × 0.25 mm, 0.25 μm; Agilent Technologies, USA). Standard fatty acid mixtures (Fame #16, RESTEK) were used as references for calibration. For fatty acid quantification, glyceryl triundecanoate (5 mg/mL) was used as the internal standard.

## 5. Conclusions

This study characterized the differential effects of drought stress imposed at seedling or flowering stages on maize. The WRKY and bHLH families were key transcription factors in maize response to drought. Lipid metabolism had a positive regulatory effect, with unsaturated fatty acids maintaining cell oxidative homeostasis and inducing JA signaling. The α-linolenic acid may have differential patterns of response to DS in maize seedling and flowering stages. In conclusion, the integration of metabolomic and transcriptomic approaches provides new insights into plant responses to DS.

## Figures and Tables

**Table 1:** Statistics of differentially abundant metabolites.

Group Name	All Significantly Different	Downregulated	Upregulated
CK_vs_T1	302	75	227
CK_vs_T2	294	92	202
T1_vs_T2	188	113	75

**Table 2:** Quantitative determination of α-linolenic acid.

Treatment	Content (mg/g. FW)	Significance
CK	0.03207 ± 0.00014	-
T1	0.03934 ± 0.00019	*
T2	0.04560 ± 0.00036	*

Note: Each treatment has three replicates. Statistical analysis was performed by one-way ANOVA (Software: Graphpad prime 8.0.1, GraphPad, San Diego, CA, USA). The symbol “*” indicates a statistically significant difference between CK and other treatments.

## Data Availability

The data presented in this study are available in Supplementary Material.

## Supplementary Materials

The following supporting information can be downloaded, Table S1: All Metabolites in T1 and T2. Table S2: Different Accumulated Metabolites in T1 and T2. Table S3: All Sample Summary. Table S4: All differential gene expression and annotation information. Table S5: Enrichment of DEGs pathways in T1 and T2. Table S6: Enrichment of Special DEGs pathways between T1 and T2. Table S7: Different expression transcription factors between T1 and T2. Table S8: Correlation between genes and metabolites in T1 and T2. Table S9: KEGG pathways enrichment of DEGs and DAMs between T1 and T2. Table S10: Primer design of qRT-PCR.

## Sample Availability

Samples of the compounds are not available from the authors.

## Abbreviations

DS	Drought stress; DEG: Differentially expressed gene
DAM	Differential accumulated metabolite
KEGG	Kyoto Encyclopaedia of Genes and Genomes
CP	Crude protein
SS	Soluble sugar
MDA	Malondialdehyde
JA	Jasmonic acid
